# Crowned Dens Syndrome: A Rare Complication of Calcium Pyrophosphate Crystal Deposition Disease

**DOI:** 10.7759/cureus.25593

**Published:** 2022-06-02

**Authors:** Thales Nogueira Gomes, Mariana Camelo Pereira, Alana Pinheiro Alves, Marcos Madeiro

**Affiliations:** 1 Internal Medicine, Western Michigan University Homer Stryker M.D. School of Medicine, Kalamazoo, USA; 2 Internal Medicine, Bronson Methodist Hospital, Kalamazoo, USA

**Keywords:** crystal arthropathies, inflammatory arthropathy, inflammatory neck pain, calcium pyrophosphate dihydrate crystal deposition disease, crowned dens syndrome

## Abstract

Crowned dens syndrome (CDS) is a clinical entity characterized by neck pain associated with fever, headaches, and neck stiffness, along with radiologic evidence of peri-odontoid process calcification in a characteristic “crown” or “halo” distribution. It is likely an underdiagnosed condition and patients can initially be misdiagnosed, leading to costly evaluation and unnecessary treatment interventions. We present the case of a 76-year-old man who presented to the emergency department (ED) with a 3-day history of progressively worsening neck pain that was associated with headaches, malaise, decreased oral intake, chills, and fever. Initial evaluation was significant for the presence of fever, tachycardia, and elevated inflammatory markers. We report a case of CDS attributed to calcium pyrophosphate deposition and review the pertinent literature about the presentation, diagnostic evaluation, and treatment of this rare clinical entity.

## Introduction

Crowned dens syndrome (CDS) is a rare form of crystal deposition disease that was initially described in 1985 as a clinic-radiological condition caused by microcrystalline calcium pyrophosphate or hydroxyapatite buildup in areas surrounding the odontoid process, also known as dens [1]. Clinically, it typically presents with a triad of fever, neck pain or stiffness, and headaches, with a very similar presentation to meningitis. Neurological examination is normal unless patients have severe presentations with associated cervical spine and nerve root compression [2,3]. It appears to be more common in women and usually affects elderly patients, with one study finding average ages of 69 for men and 75 for women [4]. It can present acutely or sub-acutely. Since symptoms are non-specific, patients usually undergo workups for other more common or life-threatening processes such as bacterial meningitis or cervical spondylitis. In one case series, eight patients had atypical presentation of CDS mimicking giant cell arteritis (shoulder pain and jaw claudication), polymyalgia rheumatica, meningitis, or discitis [5]. Biochemically, CDS features elevated inflammatory markers such as C-reactive protein (CRP) and erythrocyte sedimentation rate (ESR), in addition to a slight elevation of WBC count [2]. It is likely an underdiagnosed condition and patients are often initially misdiagnosed, leading to costly evaluation and unnecessary treatment interventions. In this article, we present a case of acute CDS initially treated as meningitis in an elderly man.

## Case presentation

Our patient is a 76-year-old man with a past medical history of hypertension, hyperlipidemia, and stroke who presented to the emergency department (ED) for evaluation of progressively worsening neck pain that started 3 days prior. He described it as aching/throbbing in nature and it was associated with limited neck mobility, headaches, malaise, decreased oral intake, chills, and subjective fever. A review of systems was negative for photophobia, phonophobia, nausea, vomiting, skin rash, confusion, aphasia, seizures, or myalgias.

**Figure 1:**
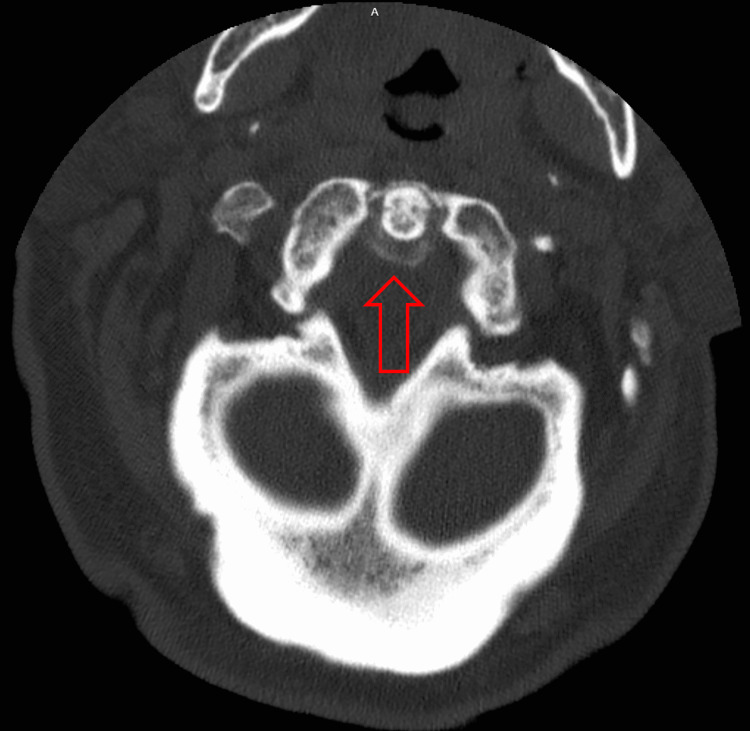
CT neck axial view demonstrating area of calcification around the odontoid process (red arrow).

**Figure 2:**
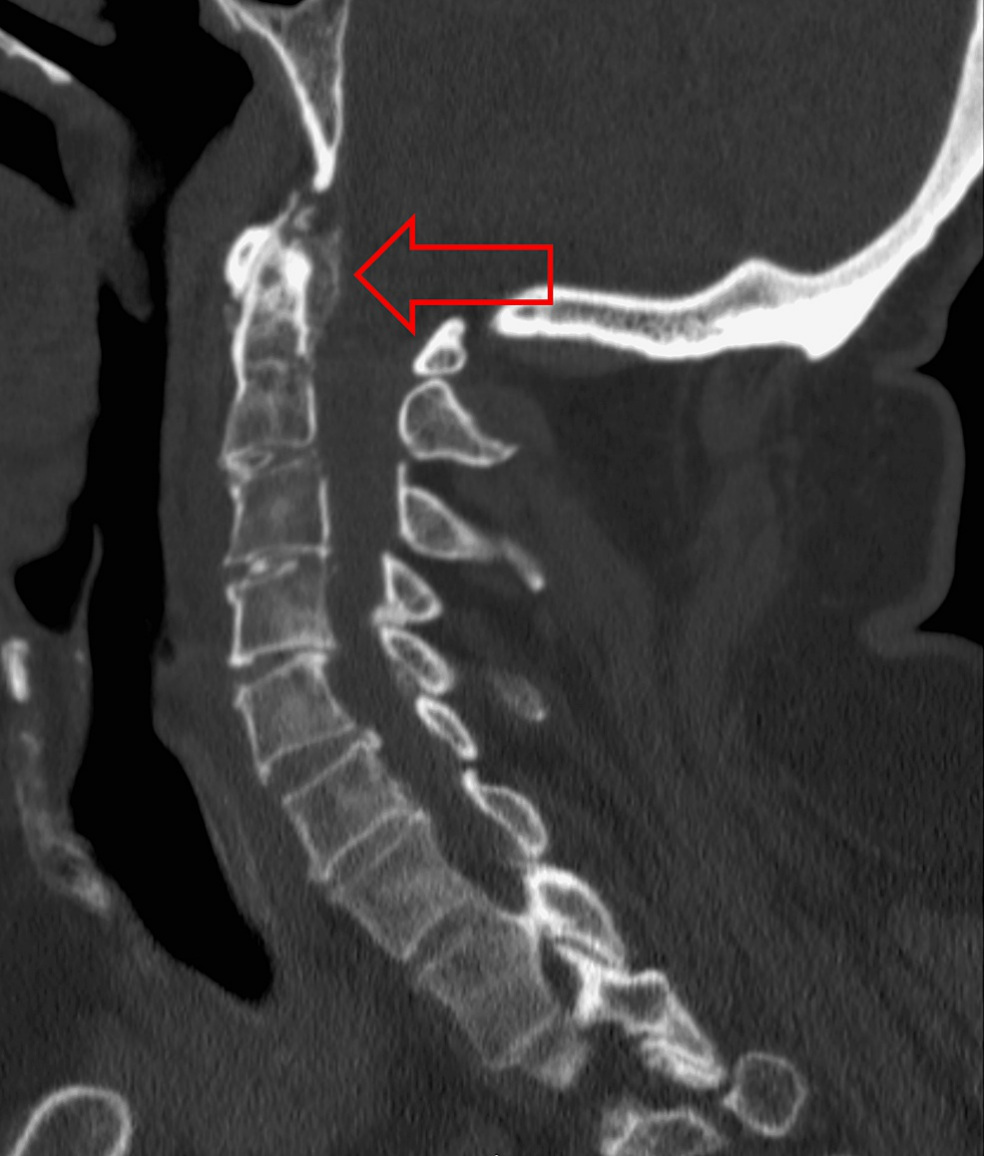
CT neck sagittal view showing area of calcification posterior to the odontoid process (red arrow).

In the ED, vital signs were significant for a temperature of 100.7°Fahrenheit, pulse of 105, and blood pressure of 164/72 mmHg. Physical exam was significant for limited range of motion of the neck due to pain, but Kernig’s and Brudzinski’s signs were negative. No papilledema was seen on fundoscopy. Mental status and neurologic exam were normal. Computerized tomography (CT) head without contrast revealed no acute findings. Due to concerns for meningitis, a lumbar puncture was performed, and the patient was started on empiric vancomycin and ceftriaxone. However, cerebrospinal fluid (CSF) evaluation was reassuring, consisting of four nucleated cells (reference range 0-5/µL), clear appearance, glucose of 89, and minimal elevation of protein (76 mg/dL-reference range 15 - 45 mg/dL). Inflammatory markers were elevated, with CRP of 119.6 mg/L (reference range < 6.0 mg/L) and ESR of 28 mm/h (reference range <20 mm/h). Given our patient’s non-toxic appearance and the benign CSF findings, CDS was suspected. A dedicated CT scan of the neck was obtained, revealing a rim of calcification around the odontoid process, supporting the diagnosis of CDS (Figures 1-2).

**Figure 3:**
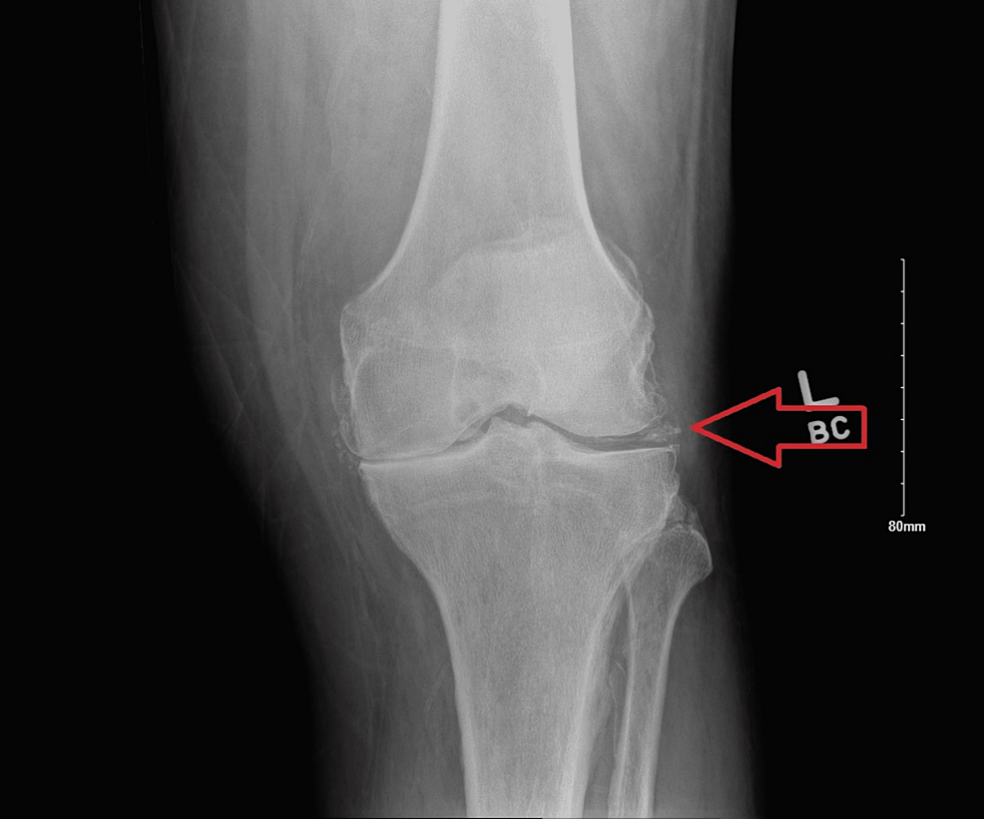
Left knee X-ray anteroposterior incidence showing signs of osteoarthritis (joint space narrowing and osteophyte formation) and chondrocalcinosis (red arrow).

The patient’s hospital course was complicated by the development of acute synovitis in both knees. Left knee radiography showed signs of osteoarthritis and chondrocalcinosis (Figure 3). Arthrocentesis was performed, and synovial fluid analysis showed positively birefringent crystals consistent with calcium pyrophosphate deposition (CPPD) arthropathy. Given initial presentation with fever, neck pain, and stiffness, bacterial meningitis was the major initial diagnostic consideration. However, with the reassuring CSF analysis, neck imaging showing the rim of calcification around the odontoid process, and development of CPPD arthropathy during the hospitalization, the diagnosis of CDS was favored. Radiology input was also crucial to establishing the diagnosis.

He was eventually discharged home on a 5-day steroid taper. On outpatient follow-up 2 months later, his neck pain and stiffness had almost completely resolved.

## Discussion

CDS is a clinical entity characterized by neck pain associated with fever, headaches, and neck stiffness, along with radiologic evidence of peri-odontoid process calcification in a characteristic “crown” or “halo” distribution [2]. Diagnostic evaluation can be challenging since the clinical picture can be indistinguishable from that of meningitis. Alternatively, when there is pain involving the shoulders or temporomandibular joints, CDS may closely resemble giant cell arteritis or polymyalgia rheumatica [5].

In our case, the initial diagnosis of meningitis was ruled out by a reassuring CSF analysis and cultures, leading to further diagnostic evaluation with a CT of the neck that was able to identify the characteristic crown-shaped or horseshoe-like calcifications around the dens. It should be noted, however, that the presence of calcification around the dens is not pathognomonic for CDS, since its diagnosis also requires a matching clinical picture [6]. CT focusing on C1-C2 is the gold standard for diagnosis of CDS and MRI should be reserved for when there are concerns for neurologic complications. Most patients will have symmetric posterior deposition of crystals around the dens, but deposits could be lateral, postero-lateral, or anterior, anywhere in the synovial membrane, transverse ligament, articular capsule, transverse cruciate, and alar ligaments [2,5].

It is important that imaging is obtained during an acute attack of CDS, as CT performed after calcifications have been reabsorbed can fail to detect this condition. For this reason, repeating imaging is recommended [7].

Interestingly, our patient developed acute pseudogout of bilateral knees concomitantly with neck pain, which corroborated the diagnosis of CDS. This finding is supported by the literature, as an extensive review of 40 patients with CDS showed that over half the patients diagnosed with this condition had a past medical history of pseudogout attacks in other anatomical locations, and 65% had previous articular chondrocalcinosis in the knee, wrist, ligamentum flavum of the cervical spine, or pubic symphysis [2]. Although plain radiographs of the neck do not aid in identifying crystal deposition on the odontoid process, obtaining X-rays of knees and wrists has been suggested as a helpful approach in diagnosing CDS, even if patients are not manifesting arthralgia or synovitis in those joints [5].

Risk factors for CPPD include advanced age, presence of osteoarthritis, low cortical bone mineral density, end-stage renal disease, and primary hyperparathyroidism, among others [8]. The pathophysiology of calcium pyrophosphate or hydroxyapatite deposition is not completely elucidated. Previous studies show that crystals can induce a sterile inflammation through increasing secretion of interleukins (such as IL-6 and IL-8) by synovial lining cells, plus prostaglandin E, and other pro-inflammatory agents [9]. An in-vitro cell study revealed that pyrophosphate crystals in the joint will produce an analogous response to chondrocytes and synoviocytes extracted from human cells of osteoarthritic knees, which can induce the release of catabolic mediators and exert multiple pathogenic effects [10].

There is no directed pharmacological therapy to eliminate the crystal deposits present in the joints or to prevent their deposition, therefore treatment is focused on reducing associated inflammation. The mainstay therapy for CDS is similar to that of other crystal arthropathies, consisting of non-steroidal inflammatory drugs (NSAIDs) and low-dose steroids; this approach typically leads to dramatic symptom improvement, as was the case for our patient [11]. Low-dose colchicine has been suggested as an option for long-term treatment in case CDS presents in a subacute or recurrent fashion [12]. For the rare cases in which CDS results in cervical myelopathy with neurological decline, surgical decompression, and stabilization is the main approach to treatment [2].

## Conclusions

CDS is a rare clinical entity that should be considered as a possibility in patients presenting with neck pain, headache, and fevers, especially when bacterial meningitis workup is negative and there is a history of CPPD arthropathy. CT of the neck is a fundamental tool for an accurate diagnosis of CDS, with the classic finding of a rim of calcification around the odontoid process. There have been no large clinical studies concerning appropriate treatment for this condition. However, data from case reports suggest NSAIDs and oral steroids are usually associated with favorable outcomes and symptom improvement, as evidenced in our case.
